# Understanding the potential and challenges of adenoma treatment as a prevention opportunity: Insights from the BeWEL formative study

**DOI:** 10.1016/j.ypmed.2011.10.017

**Published:** 2012-01-01

**Authors:** Martine Stead, Stephen Caswell, Angela M. Craigie, Douglas Eadie, Annie S. Anderson, The BeWEL team

**Affiliations:** a Institute for Social Marketing, Stirling Management School, University of Stirling and The Open University, Stirling, FK9 4LA, UK; b Centre for Research into Cancer Prevention and Screening, Division of Clinical & Population Sciences & Education, University of Dundee, Dundee, DD1 9SY, UK

**Keywords:** Qualitative research, Focus groups, Cancer screening, Adenoma, Polyps, Lifestyle, Intervention, Prevention

## Abstract

**Objectives:**

To explore prevention opportunities presented by colorectal adenoma diagnosis and inform engagement strategies for the BeWEL study (body weight and physical activity lifestyle intervention for colorectal cancer screening participants who have undergone adenoma removal).

**Methods:**

Qualitative study comprising 4 purposively sampled focus groups conducted in urban and rural areas in Tayside, Scotland, with different deprivation levels. Participants were men and women (n = 17) aged 50-74 with BMI > 25 kg/m^2^ with removal of adenoma detected by colorectal cancer screening.

**Results:**

Adenoma diagnosis presents both opportunities and challenges for prevention. Some patients perceived adenoma as minor and not sufficiently motivating to act as a ‘teachable moment’. Patients had low awareness of the relationship between adenoma and lifestyle factors, and received little information on prevention during screening and treatment. Consequently they interpreted post-treatment ‘all clear’ messages as validation of existing lifestyles, and did not see the relevance of prevention advice. Receptiveness increased when the association between lifestyle, adenoma recurrence and other illness was explained.

**Conclusion:**

The study illustrates the value of exploratory research into patient understanding to improve communications and health services. Without unduly worrying patients, professionals should explain how to reduce risk of adenoma, cancer and other diseases, particularly through diet, physical activity and weight reduction.

## Highlights

► Adenoma diagnosis presents opportunities and challenges for prevention. ► Screening patients did not appear to know that the polyps could be pre-cancerous. ► Awareness of the role of lifestyle in preventing adenoma recurrence was limited. ► Screening professionals should endorse adenoma prevention messages.

## Introduction

Colorectal cancer (CRC) is the third most common cancer and cause of cancer death in the USA and UK (IARC, 2010). Most cases (95%) occur in people over 50 years, often co-existing with other lifestyle-related diseases including type 2 diabetes mellitus and cardiovascular disease (CVD) (Baade et al., 2006; Brown et al., 1993). These diseases share common risk factors including large body size, abnormal lipids and markers of insulin resistance (Giovannucci, 2007). The UK government strategy aimed at decreasing CRC burden is focussed on early detection of the disease, and national CRC screening programmes using faecal occult blood testing (FOBT) have been rolled out across the UK (www.cancerscreening.nhs.uk/bowel).

A positive result from screening can focus participants' attention on risk reduction (McBride et al., 2008), and intervention studies have demonstrated a positive response to dietary guidance (Baker and Wardle, 2002; Caswell et al., 2009; Robb et al., 2010). However, screening also has the potential to provide false reassurance – the ‘health certificate’ effect, whereby patients who receive negative results feel no need to modify their lifestyle, or have poorer health behaviours than those not participating in screening (Larsen et al., 2007). Both these potential consequences of screening underline the importance of understanding perceptions about disease causes and lifestyle factors, and how these might shape response to prevention interventions. Messages and advice given by professionals during screening are likely to influence how people interpret and respond to results and treatment, particularly in relation to making subsequent health behaviour changes (Miles et al., 2010).

The work reported here was undertaken as part of formative research to gather insight into patients' perspectives about lifestyle interventions after receiving a positive CRC screening result. This study was then utilised to inform thinking about recruitment and intervention approaches for the BeWEL study – a randomised controlled trial (RCT), designed to measure the impact of a body weight and physical activity intervention on adults at risk of developing colorectal adenomas (Craigie et al., 2011). The focus of the BeWEL intervention is based on evidence of an association between physical activity, obesity, and diet and risk of CRC and other chronic diseases (Knowler et al., 2002; WCRF, 2007), and that approximately 43% of CRC can be prevented through changes in these risk factors (WCRF, 2009).

The current work, undertaken before recruitment to the full BeWEL study, explored how participants with adenoma detected and removed through the CRC screening programme felt about their diagnosis, their understanding of its significance, and the extent to which the experience might motivate behaviour changes to reduce CRC and other chronic disease risk. The study also explored whether adenoma diagnosis might represent a ‘teachable moment’ (Lawson and Flockie, 2009), and how this moment might be better utilised as a prevention opportunity.

## Methods

### Study participants

Prospective participants aged 50–74 and living within Tayside, Scotland, who had undergone adenoma removal within the last three months were identified retrospectively from hospital records and invited to participate in a focus group. All patients were advised of the study through a letter of introduction sent by the colorectal nurse specialist responsible for screening. This letter was then followed two weeks later by a written invitation from the research team. Those interested were telephone screened for BMI (> 25 kg/m^2^) and availability. Recruitment was from a mix of urban and rural populations and a range of social backgrounds, as assessed by the Scottish Index of Multiple Deprivation (SIMD) which defines deprivation at the postcode level on the basis of income, employment, health, education, skills, housing, geographical access and crime (Scottish Government, 2009). Written informed consent was obtained prior to the focus groups.

### Data collection

A discussion guide was developed containing open-ended questions around key areas including experiences of adenoma diagnosis and treatment, understanding of adenoma and its relationship to lifestyle and disease, and how participants would feel about being offered advice and support for making behaviour changes, particularly in relation to healthy eating, physical activity and weight loss. Focus groups were moderated by an experienced researcher and digitally audio-recorded with participants' consent.

### Data analysis

Recorded discussions were transcribed and a thematic analysis was conducted. The approach drew on both the deductive and inductive approaches to thematic analysis (Braun and Clarke, 2006): themes relating to the pre-specified research questions (for example, attitudes towards receiving lifestyle advice) were actively sought in the data, whilst further themes evolved from the coding process itself (for example, the perceived contradiction between receiving an all-clear message during screening and then being offered advice for lifestyle change).

Ethical approval was given by NHS Tayside's Committee on Medical Research Ethics.

## Results

**Fig. 1:**
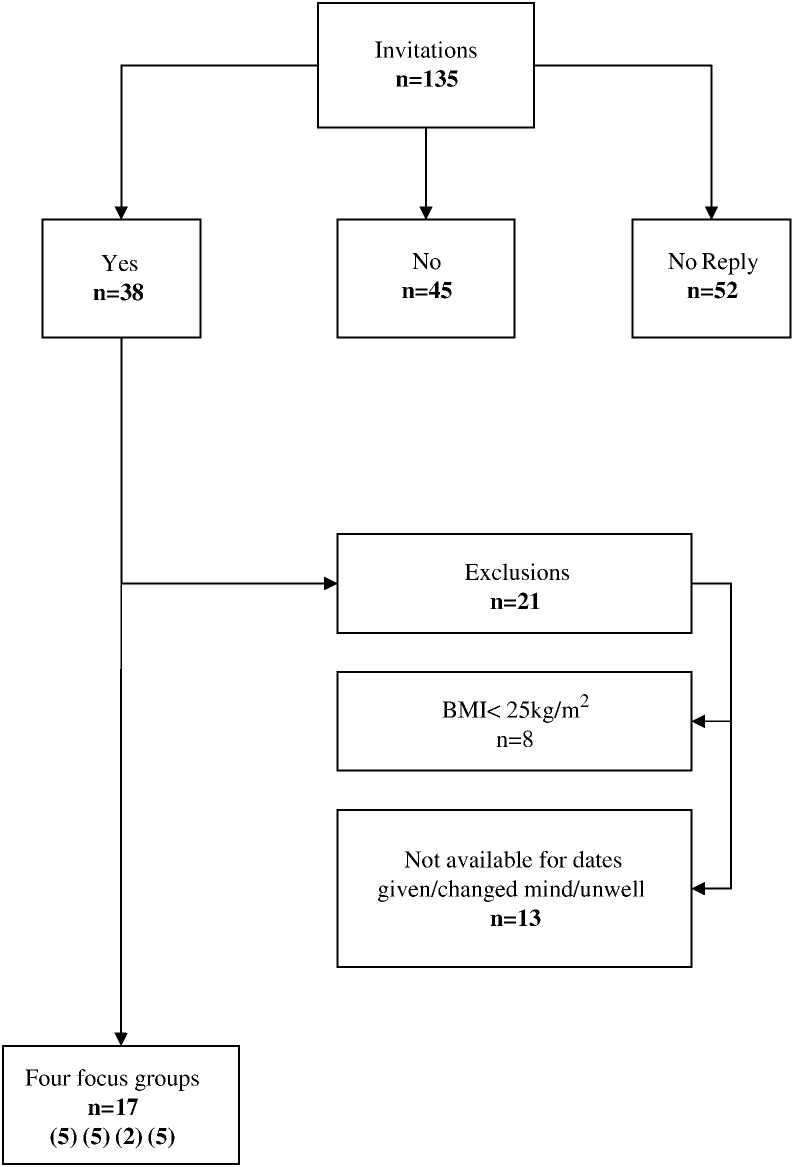
Flow diagram of recruitment to focus groups (Tayside Scotland, May to September 2010).

In total, 135 men and women were invited to take part. CRC screening nurses provided a list of the most recent 105 eligible participants, 31 females and 74 males, of whom 8 females and 22 males agreed to be contacted. A further 30 were subsequently invited, including purposive over-sampling of females to improve representation of women in the study. Of these 135, 38 agreed to be contacted. However 8 were excluded at telephone screening (self-reported BMI < 25 kg/m^2^) and 13 unavailable during the fieldwork period (Fig. 1).

Participants were male (n = 12) and female (n = 5), aged 50–74, of mixed social class and many were retired (Table 1). We conducted four focus groups: one all male and three mixed gender. Two were held in the community, two in university settings. The groups lasted between 75 and 100 min.

Reported health status and experiences varied within the focus groups and reflected the range of diseases common in this age group including CVD. Gender and SIMD were similar in participants and non-participants. We did not have information on the health or weight status of non-participants to enable comparison of these factors (Table 1).

### Experiences of adenoma diagnosis and treatment

Whilst for some participants receiving news of a positive FOBt was a shock, there was a general perception that adenoma was a minor abnormality, with concern tending to focus on the preparation for colonoscopy rather than on the possibility that adenoma could signify a major health problem.

Despite adenoma diagnosis being as a result of the CRC screening programme and colonoscopy procedures, several did not appear to know that the polyps could be pre-cancerous. Some participants only became fully aware of this in discussion with others or during the focus groups. The failure to link adenoma with potential cancer appeared to be reinforced by interactions with professionals during the treatment process, which, in participants' accounts, had tended to focus on reassurance and to downplay or omit the mention of cancer.

### Understanding of adenoma causation and prevention

**Fig. 2:**
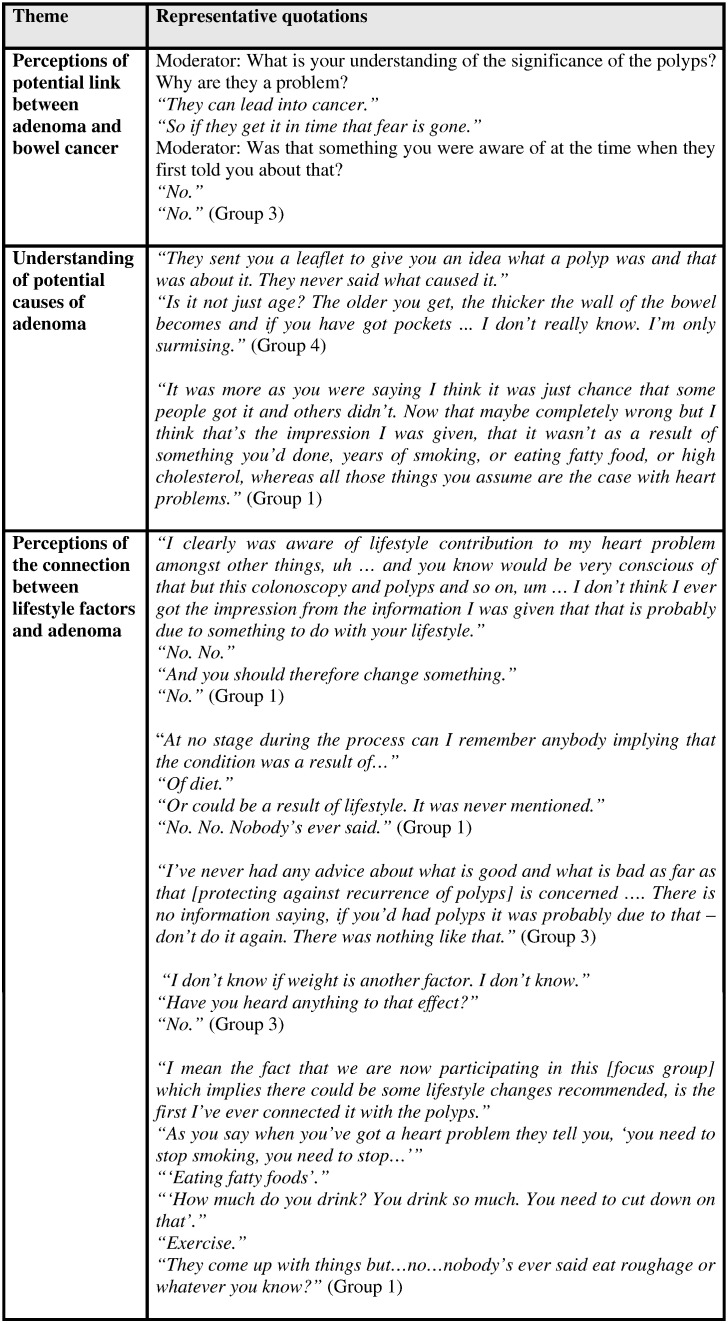
Key findings and selected representative quotations: understanding of the significance and causes of adenoma (Tayside Scotland, May to September 2010).

Participants seldom considered what might have caused an adenoma, with most saying they “didn't know”. Some ventured possible explanations, including age, genetics and “just chance”, but none recalled receiving information on possible contributory factors during the diagnosis and treatment process (see Fig. 2). Similarly, participants could not recall receiving advice during or after treatment on prevention of adenoma recurrence.

### Attitudes towards the concept of receiving lifestyle change advice following adenoma

Due to the lack of understanding of adenoma causation and prevention, the concept of receiving advice and support for lifestyle change following adenoma treatment initially appeared to make little sense. Participants were not encouraged to think about prevention during the treatment process, either in relation to adenoma specifically or more widely.

**Fig. 3:**
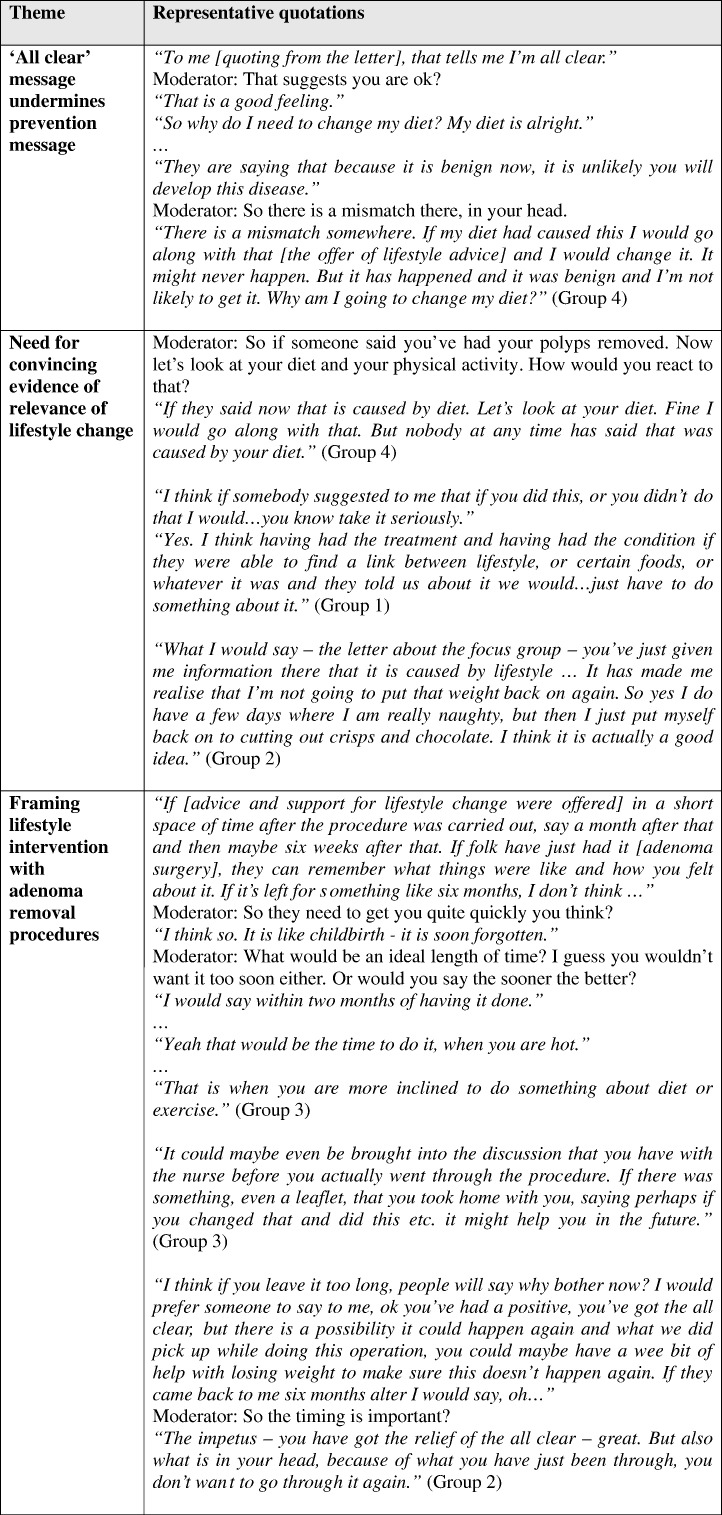
Key findings and selected representative quotations: response to concept of being offered lifestyle change advice following adenoma treatment (Tayside Scotland, May to September 2010).

Furthermore, some of the information participants received contradicted the idea that prevention was important (Fig. 3). The reassuring ‘all clear’ messages participants received post-treatment, from verbal and written communications with health professionals, implied a “clean bill of health”, indicating there was nothing about their current lifestyle requiring modification. Some quoted in this context from the focus group invitation letter, which emphasised to invitees that their adenoma was successfully treated and they were unlikely to develop bowel cancer:

“To me, that tells me I'm all clear… so why do I need to change my diet?” (Group 4).

This apparent contradiction contributed to scepticism and defensiveness in relation to the concept of receiving lifestyle advice following adenoma treatment. Some described themselves as “unconvinced” of a connection between lifestyle, adenoma and bowel cancer, and needed persuading of a potential causal link between their own behaviour and the condition before they would consider making lifestyle changes (Fig. 3). Some suspected that the adenoma treatment process might be used simply to promulgate ‘correct’ lifestyle advice to a captive group “just because it is the done thing” (Group 4), rather than because adenoma patients were specifically in need of lifestyle change.

This scepticism was expressed against a backdrop of wider ambivalence about lifestyle change. A few were dismissive, regarding lifestyle advice as inconsistent and arbitrary — “if you read the newspapers you realise that whatever you do is bad for you!” (Group 1). Others felt that the possibility of change was unrealistic “at our age” (Group 1), particularly in relation to weight loss which was perceived to be more difficult as one became older and the “pace of life” slowed (Group 3).

### Utilising the teachable moment

More positively, some welcomed the possibility of help to address aspects of lifestyle, once they grasped the notion that lifestyle factors could have contributed to their adenoma. One suggested that “the relief of the all clear” (Group 2) combined with a health professional warning them “you could maybe have a wee bit of help with losing weight to make sure this doesn't happen again” (Group 2) could spur someone to consider making lifestyle changes (Fig. 3).

A few said they “would be very open to suggestions about lifestyle changes” (Group 1) and receptive to being offered active support. Some commented that the timing of any lifestyle change messages was important – that information and support would need to be offered soon after adenoma treatment, whilst recollections of the procedures were still “hot” (Group 3) (Fig. 3).

## Discussion

With surveillance colonoscopy (offered to all patients with adenomas), subsequent adenomas can be identified and removed before they progress to CRC. However, colonoscopy may still miss lesions, and there have been reports of interval cancers diagnosed between examinations (Leung et al., 2010; Robertson et al., 2005). Weight gain is associated with the development of adenomas and recurrence, whilst weight loss is associated with reduced adenoma prevalence and recurrence rates (Sedjo et al., 2007; Yamaji et al., 2008). Therefore, it would seem prudent to recommend weight loss to overweight adults who have experienced an adenoma in order to minimise risk of colorectal cancers as well as related co-morbidities (Avenell et al., 2004).

This small qualitative study added to our understanding of the potential and challenges of adenoma diagnosis and treatment as a prevention opportunity and yielded insight into how patients might respond to an invitation to participate in the BeWEL RCT. The current work confirms the value, prior to the implementation of any full-scale intervention or service, of investing in exploratory research to understand the perspective of the intended target group. Such research can yield insight into patients' interpretation of health and trial information (Paramasivan et al., 2011; Stead et al., 2005), and can be used to improve communications; for example, ‘consumer insight’ research was used to inform the strategy of a social marketing media campaign in Scotland to increase awareness of bowel and oral cancer symptoms among lower socio-economic groups (Eadie and MacAskill, 2007; Eadie et al., 2009).

The current findings are limited by the sample size and by self-selection: people who agree to participate in focus groups may be more engaged in health issues and more well-disposed towards health research than the general population. Recruitment to the focus groups was lower than expected, possibly because some invitees did not wish to discuss in group settings their experiences. It is also possible that some were deterred by the allusions in the letter to making lifestyle changes. This may have implications for the BeWEL intervention study, although previous lifestyle intervention studies (Baker and Wardle, 2002; Caswell et al., 2009; Robb et al., 2010) did succeed in recruitment targets (although none focussed on weight loss).

The results also suggest that the experience of a positive FOBT and subsequent treatment might represent a ‘teachable moment’ for prevention advice in relation to CRC and other obesity related conditions (McBride et al., 2008). Encouragingly, respondents in this study were mostly positive about the screening and treatment programme, and it is possible that this may make them well disposed to attend to information and lifestyle advice offered as part of that process.

However, if adenoma diagnosis and treatment is to be a teachable moment, patients need to be aware of the risk factors for adenoma and to relate these to personal behaviours. Unlike other teachable moments, where there is a shared and accepted understanding of the relationship between disease and behaviour (e.g. lung cancer and smoking), no such link was present in participants' minds between adenoma and lifestyle. This limited awareness of the potential relationship between lifestyle factors and CRC has been reported elsewhere (Caswell et al., 2008), even among cancer survivors (Demark-Wahnefried et al., 2005). Current findings suggest that, for many, adenoma diagnosis may not trigger sufficiently strong emotional responses or increase expectations of negative outcomes to motivate behaviour change. This is partly because, for the group most likely to have adenoma detected through CRC screening, polyps are seen as a relatively minor problem compared with more serious health problems such as CVD. These low risk perceptions were reinforced by the reassuring tone of interactions during the treatment process and the downplaying of risk. For prevention advice to make sense and be motivating to CRC screening patients, the links between adenoma, CRC and lifestyle factors need to be made consistently in the screening and treatment process. The reassurance offered by professionals during these processes combined with subsequent ‘all clear’ messages can be interpreted by patients as a validation of existing lifestyles, and may reduce the credibility and salience of subsequent lifestyle advice. It would be desirable for professionals to alert people to further action that can be made to reduce risk, highlighting current evidence, suggesting simple ways to assess health behaviour, and signposting sources of advice and support.

**Fig. 4:**
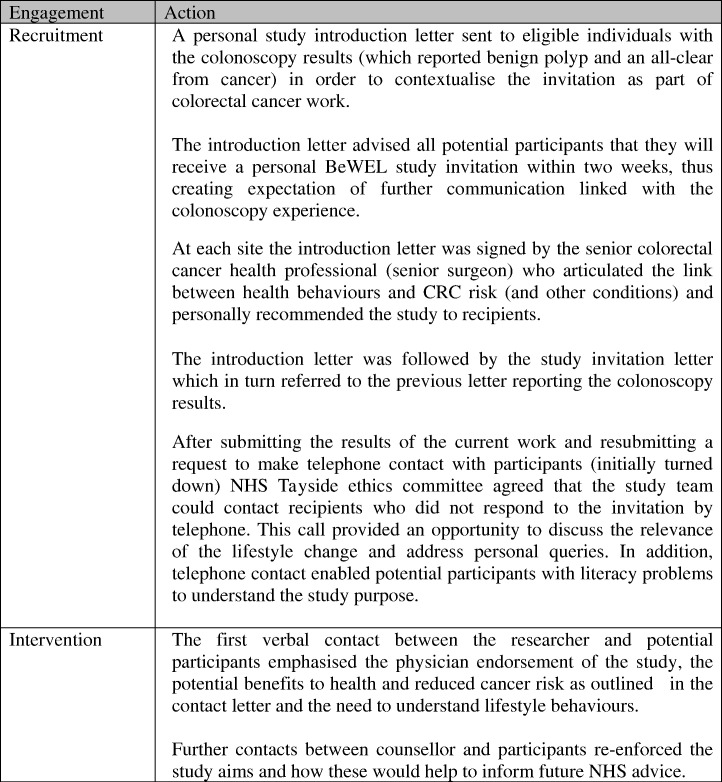
Strategies undertaken to assist participation and recruitment to BeWEL RCT resulting from the BeWEL focus group work.

The study has identified helpful learning points for the recruitment and intervention protocol of the full BeWEL RCT (Fig. 4). It suggests that CRC health professionals should act as advocates for lifestyle change and promotion of the study. The findings raise the possibility that written information about the study will be the first time recipients learn of an explicit connection between lifestyle and CRC, and this could be usefully reinforced, especially with people who do not respond to the study invitation. For people who express interest in the study and are recruited, researchers could repeat the endorsement of the study by the lead clinician. Importantly, health professionals and researchers need to encourage participants to look ahead to opportunities for health gain, avoiding any sense of victim blaming for cancer risk (Chapple et al., 2004), whilst motivating and supporting lifestyle change for the years ahead.

## Conflict of interest statement

All authors have completed the Conflict of interest policy form and declare: no support from any organisation for the submitted work; no financial relationships with any organisations that might have an interest in the submitted work and, no other relationships or activities that could appear to have influenced the submitted work.

## Figures and Tables

**Table 1:** Participant and non-participant characteristics (Tayside May to September 2010).

	Invitations (n = 135)	Participants (n = 17)	Non participants			
No (n = 45)	No reply (n = 52)	Excluded: BMI < 25 kg/m^a^ (n = 8)	Unavailable^a^ (n = 13)
Male (%)	88 (65)	12 (71)	26 (58)	35 (67)	7 (88)	8 (62)
Female (%)	47 (35)	5 (29)	19 (42)	17 (33)	1 (12)	5 (38)
SIMD^b^ deciles 1–3 (%)	37 (27)	3 (18)	13 (29)	18 (35)	2 (25)	1 (8)
SIMD^b^ deciles 4–7 (%)	59 (34)	8 (47)	20 (44)	20 (38)	5 (63)	6 (46)
SIMD^b^ deciles (8–10%)	39 (29)	6 (35)	12 (27)	14 (27)	1 (12)	6 (46)

a Not available for dates given/changed mind/unwell.

b SIMD Scottish Index of Multiple Deprivation, deciles 1–3 most deprived, 8–10 least deprived.
